# Low-Power and Eco-Friendly Temperature Sensor Based on Gelatin Nanocomposite

**DOI:** 10.3390/nano12132227

**Published:** 2022-06-29

**Authors:** Giovanni Landi, Veronica Granata, Roberto Germano, Sergio Pagano, Carlo Barone

**Affiliations:** 1 ENEA, Casaccia Research Center, Via Anguillarese 301, 00123 Rome, Italy; 2 Dipartimento di Fisica “E.R. Caianiello”, Università degli Studi di Salerno, 84084 Fisciano, Italy; vgranata@unisa.it (V.G.); spagano@unisa.it (S.P.); 3 INFN Gruppo Collegato di Salerno, c/o Università degli Studi di Salerno, 84084 Fisciano, Italy; 4 PROMETE Srl, CNR Spin off, P.le V. Tecchio, 45, 80125 Naples, Italy; germano@promete.it; 5 CNR-SPIN, c/o Università degli Studi di Salerno, 84084 Fisciano, Italy

**Keywords:** temperature sensor, sustainability, environmental monitoring, gel polymer electrolyte, gelatin, graphene, water processable, self-powered, current limiting phenomena, faradaic process, energy efficiency

## Abstract

An environmentally-friendly temperature sensor has been fabricated by using a low-cost water-processable nanocomposite material based on gelatin and graphene. The temperature dependence of the electrochemical properties has been investigated by using cyclic voltammetry, chronopotentiometry and impedance spectroscopy measurements. The simple symmetric device, composed of a sandwich structure between two metal foils and a printable graphene–gelatin blend, exhibits a dependence on the open-circuit voltage in a range between 260 and 310 K. Additionally, at subzero temperature, the device is able to detect the ice/frost formation. The thermally-induced phenomena occur at the electrode/gel interface with a bias current of a few tens of μA. The occurrence of dissociation reactions within the sensor causes limiting-current phenomena in the gelatin electrolyte. A detailed model describing the charge carrier accumulation, the faradaic charge transfer and diffusion processes within the device under the current-controlled has been proposed. In order to increase the cycle stability of the temperature sensor and reduce its voltage drift and offset of the output electrical signal, a driving circuit has been designed. The eco-friendly sensor shows a temperature sensitivity of about −19 mV/K, long-term stability, fast response and low-power consumption in the range of microwatts suitable for environmental monitoring for indoor applications.

## 1. Introduction

Among environmental parameters, temperature is a decisive quantity for monitoring the health and thermal comfort of humans and for different industrial processes. In the literature, temperature sensors, based on metals, semiconducting, polymer and biodegradable materials, are widely reported and can be bolometers, thermistors, diodes, thermoelectric/thermocouples or resistive devices [1,2,3]. In the last decade, the research activity has put considerable effort into improving the environmental profile of electronic devices primarily by using more sustainable materials, and also by optimizing their energy efficiency [4,5,6,7]. The use of materials with low environmental impact, or from completely renewable resources, may offer several advantages, especially eco-friendliness and ease of disposability, increased functionality, non-toxicity, increased design flexibility, and, possibly, reduced unit cost [5,6]. Moreover, the reduction in the energy consumption for the electronic devices can contribute to good management of energy resources becoming a further solution for sustainability and the transition from fossil fuels [7]. Therefore, the constraints on energy performance lead us to consider devices that can be self-powered or characterized by low power consumption.

Recently, electrolytes based on hydrogel have been extensively investigated due to their conductive ionic properties and capability to form a galvanic cell with metal electrodes. Jonsson et al. reported an electrolyte-assisted temperature sensor (operating within a variation of 15 °C) based on a hydrogel with a negative voltage sensitivity of about 11 mV/K [8]. Moreover, Ortega et al. described a self-powered smart patch for sweat conductivity monitoring where the body fluid acts as the battery electrolyte [9]. Here, the presence of the galvanic cell permits to minimize completely or in part the power consumption of the device.

Among these hydrogels, gelatin is a biodegradable polymer obtained from the hydrolysis of the fibrous insoluble collagen present in bones and skin [10]. Its ability to form a hydrogel that traps a large number of liquid molecules and fillers allows it to enhance the dielectric and mechanical properties of the blends [11,12,13].

The presence of a large number of polar functional groups within the gelatin hydrogel, that can be influenced by an electric field polarization, permits the development of actuators [14], biosensors and organic transistors [15] based on gelatin. Although this hydrogel responds to environmental changes, such as pH, temperature and ionic strength, in literature, few works report the gelatin as a temperature sensor. Silva et al. described a temperature sensor in gelatin based on electrical capacitance with a sensitivity of 0.045 pF/°C at room temperature [16]. Moreover, Lan et al. developed a visual physiological environmental temperature sensor with gelatin-stabilized luminescent silver nanoclusters [17]. More recently, Qin et al. reported a gelatin organohydrogel-based temperature sensor with a broad operating temperature by adding citrate (Na_3_Cit) in the water/glycerol blend [18].

In the present study, an environmentally-friendly temperature sensor has been fabricated by using a hydrogel material based on gelatin and graphene. Since the gelatin is a weak acid, at the electrode interfaces, a potential takes place when a bias current of a few tens of μA has been applied. The occurrence of dissociation reactions within the sensor causes limiting-current phenomena in the gelatin electrolyte. Under current-controlled operation mode, the device is sensitive to temperature variations in the range between 260 K and 310 K. The mechanical stability at subzero temperatures of the sensor is obtained by the presence of glycerol, which inhibits the formation of ice crystallization [19]. A detailed model describing the charge carrier accumulation, the faradaic charge transfer and diffusion processes within the device in the current-controlled mode has been proposed. Here, the voltage sensitivity is the sum of two contributions: the first is related to the self-power component and the second is caused by the current biasing. The sensor shows a low power consumption in the range of micro-watts. In order to increase the cycle stability of the temperature sensor and reduce its voltage drift and offset of the output electrical signal, a specific driving circuit has been designed.

## 2. Materials and Methods

### 2.1. Material Preparation

Polyethylene terephthalate (PET) foil (Melinex ST 504, DuPont Teijin Films, Chester, VA, USA, thickness 125 μm) covered with copper (Cu) tape (Kohree, City of Industry, CA, USA, thickness 40 μm) was used as the substrate. Gelatin from bovine skin (type B) with a gel strength of about 225 g Bloom, glycerol and graphene nanoplatelets were purchased from Sigma-Aldrich. All aqueous solutions were prepared with Milli-Q water (18.2 MΩ cm). The gelatin blend was prepared by adding glycerol to improve its mechanical properties. The gelatin powder was dissolved in a mixture of water and glycerol at 80 °C to obtain a gelatin solution with a concentration of 10 wt.%. The graphene was added to the aqueous suspensions to give mixtures with 0.25 wt.% of filler contents. These mixtures were then mechanically stirred for about 30 min at the same temperature. The obtained gel–nanocomposite was deposited by blade coating on the PET–Cu stack and dried at room temperature. The device was completed by facing another electrode (PET–Cu foil) to form a sandwich structure. The electrode area was 2.5 × 2 cm^2^.

### 2.2. Electrochemical Measurements in the Room-Temperature Region

The electrochemical characterizations, such as cyclovoltammetry (CV) and electrochemical impedance spectroscopy (EIS), of the sample were measured on a commercial platform (Arkeo-Cicci Research srl, Grosseto, Italy) at a temperature range between 288 and 303 K. The devices were measured in a two-electrode geometry with an average area of about 5 cm^2^. The EIS measurements were performed in the frequency range between 100 mHz and 10 kHz with an ac-signal amplitude of 50 mV at open-circuit voltage. The capacitance C of the symmetric device was computed by integrating the area under the CV curves according to the following equation [20]:

(1) $$C=\frac{1}{2\cdot \nu \cdot \left(V_{b}-V_{a}\right)}\cdot \int_{V_{a}}^{V_{b}}i\left(V\right)dV,$$

where *υ* is the scan rate, *i(V)* is the charging/discharging current and *V_b_ − V_a_* is the potential window.

By assuming that the dynamic ac model of the symmetric device is a simple series connection between a resistance $R_{0}$ and a parallel branch composed of the interfacial resistance $R_{i}$ and a capacitance $C_{i}$*,* the total impedance at a given angular frequency ω can be written as

(2) $$Z=R_{0}+R_{i}/\left(1+j\omega \tau_{i}\right),$$

where $\tau_{i}=R_{i}C_{i}$. Here, the nearly pure capacitive behavior is displayed at small frequencies, where $\omega \tau_{i}\gg 1$. Therefore, the equivalent capacitance extracted from the spectra can be obtained as [21]

(3) $$C_{EIS}\approx -1/\left(\omega Z_{imag}\right),$$

where $Z_{imag}$ is the imaginary part of impedance Z. From the EIS spectra, the bulk ionic conductivity $\left(\sigma_{AC}\right)$ of the gel nanocomposite can be estimated by [22]

(4) $$\sigma_{AC}=L/\left(A\cdot Z_{real}\right),$$

where $Z_{real}$ is the real part of impedance when the phase angle goes to zero, *L* is the thickness of the gel nanocomposite and *A* is the area of the device.

### 2.3. Temperature-Dependent Transport Measurements

In order to explore the typical operating region of environmentally-friendly temperature sensors, electric transport characterizations were performed far from room temperature by using a thermoelectric cooled Peltier-type system, with a nominal range from 250 to 340 K. As already observed in the case of green electronic compounds based on sodium-alginate biopolymer [23,24], these experimental investigations are very informative to better understand the interplay between the different layers of the device sandwich structure. A LM35 sensor (Texas Instruments, Dallas, TX, USA) in contact with the sample holder was used to measure the temperature, whose stabilization was realized through a computer-controlled Proportional–Integral–Derivative (PID) loop to better than 0.2 K. This experimental setup was already successfully adopted for the measurement of conventional silicon solar cells [25,26] and innovative organic photovoltaic devices [27,28]. The samples were biased with a low-noise Keithley DC current source (Tektronix Inc., Beaverton, OR, USA) and the DC voltage drop was recorded with a digital multimeter. All the bias and readout circuitry was connected by using low-noise homemade electronics [29,30,31] and controlled by a dedicated computer equipped with the LabVIEW environment (National Instruments, Austin, TX, USA).

## 3. Results

**Figure 1:**
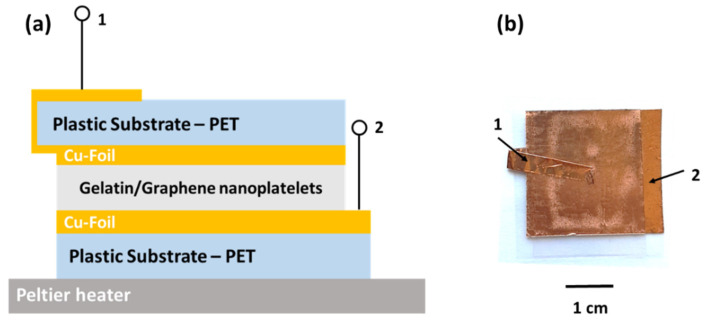
(**a**) Cross-section and (**b**) top view photograph of the symmetric device. The electrodes are indicated as (1) and (2).

Figure 1a shows the cross-section of the device formed by a symmetric sandwich structured following the layer sequence: PET substrate/Cu-Foil/Gelatin-graphene nanoplatelets. The thickness of the active layer, based on a blend of gelatin and graphene, is about 1800 µm. The gelatin/graphene system exhibits a layered and homogeneous morphology giving evidence of the good dispersion of the filler [11,12]. As reported in the literature, the 2D band shape from the Raman spectra indicates the presence of few-layer graphene flakes with an average of 10–15 atomic layers corresponding to cluster dimension in the range between 15–20 nm [11]. In Figure 1b, the image of the final device before the experimental characterization is shown. The use of a low amount of graphene as filler permits the dielectric properties of the active material to be enhanced compared to the pure gelatin, reaching an electrode specific capacitance value of about 380 F/g [11]. In particular, the conductive filler strongly influences the reaction kinetics between the electrode and the biopolymer [12]. This finding has been observed—after electrical oxidation at the anode contact occurs—within the blend when a bias voltage has been applied. It is worth noting that, to ensure an environmentally safe and easily disposable device, any ionic components that may adversely affect the environment have been avoided. Here, the gelatin acts as a binder for holding together the hydrophobic filler with the water–glycerol molecules and as a solid electrolyte when being a protonic conductor [32].

### 3.1. Temperature Dependence of Electrical Characteristics

**Figure 2:**
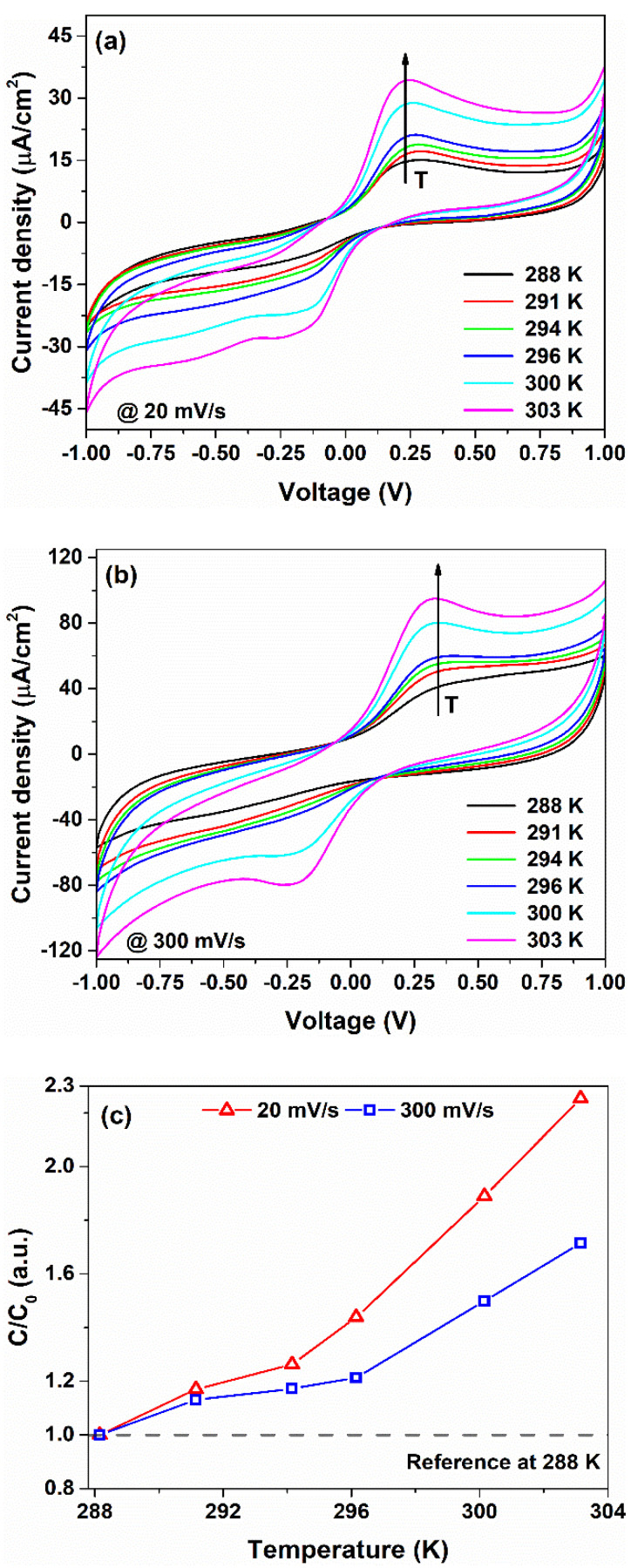
(**a**) Cyclic voltammetry curves measured in a temperature range between 288 and 303 K at (**a**) 20 and (**b**) 300 mV/s. (**c**) Temperature dependence of the normalized capacitance extracted by using Equation (1).

The temperature (T) response of the investigated electrochemical devices is influenced by the charge accumulation, faradaic charge transfer and diffusion mechanisms within the bulk and at the metal/gel nanocomposite interfaces, respectively. To highlight these contributions, cyclic voltammetry and impedance measurements have been performed in a temperature range between 288 and 303 K. Figure 2a,b shows the CV loops, measured at 20 and 300 mV/s, corresponding to a slow and fast charge carrier dynamic, respectively. For both the *υ*-values, the temperature influences the shape of the CV curves. As *T* increases, the hysteresis loop increases, suggesting that the dielectric properties of the blend are sensitive to the temperature variation ΔT. By taking into account Equation (1), the *C* values can be computed. Figure 2c shows the normalized capacitance $C/C_{0}$ in temperature for lower (20 mV/s) and higher (300 mV/s) scan-rate values. Here, $C_{0}$ is the capacitance value at 288 K (15 °C) for both the *υ*-values computed from the CV curves, respectively. As can be seen, the sample exhibits a positive temperature coefficient of the capacitance (*TCC*) defined as $TCC=\frac{1}{C_{0}}\frac{\Delta C}{\Delta T}$, where ΔC represents the capacitance variation induced by ΔT. The resulting values are 8.3 %/K and 4.6 %/K at 20 and 300 mV/s, respectively. A higher value of *TCC* observed for a lower scan rate suggests that polarization phenomena, due to the ion accumulation at the metal/gel interface, occur in the device when a temperature variation is applied along with the thickness.

**Figure 3:**
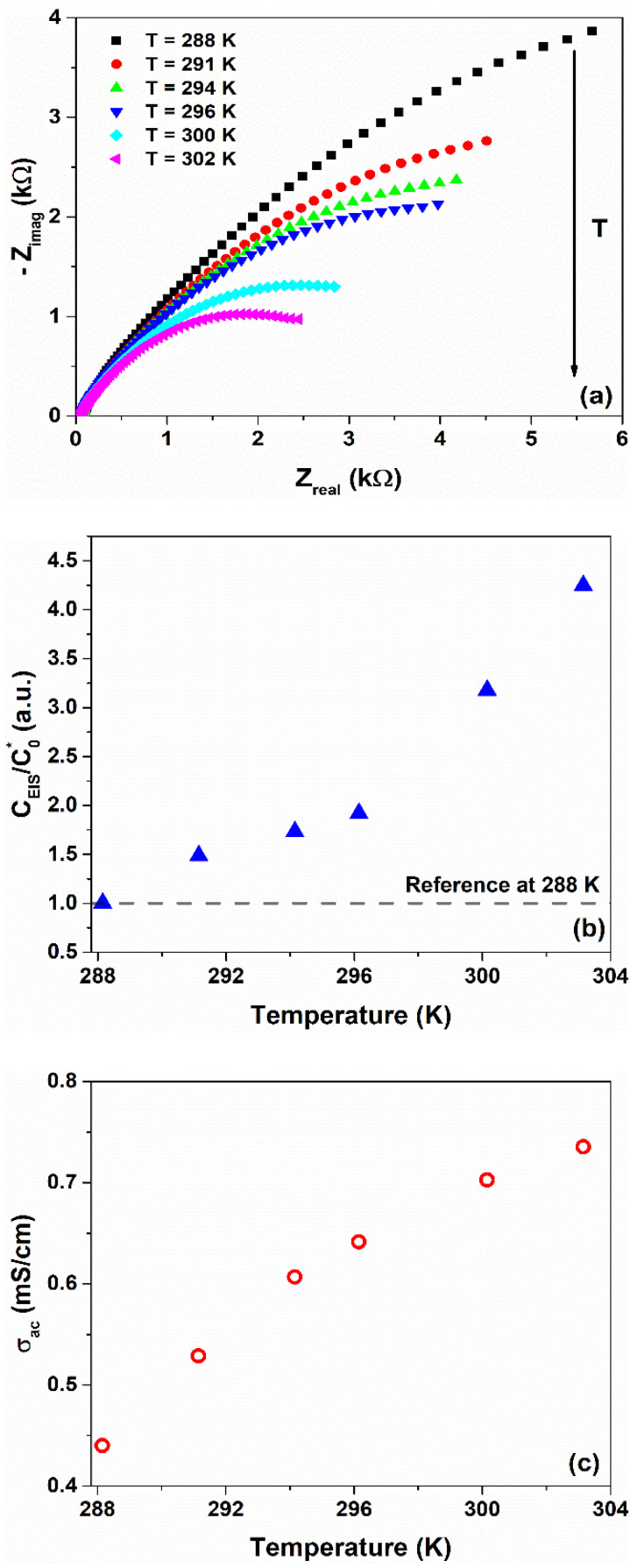
(**a**) Impedance spectra measured in a temperature range between 288 and 303 K. Temperature dependence of the (**b**) normalized capacitance and (**c**) ionic conductivity extracted from the impedance spectra by using Equations (3) and (4), respectively.

Similar to the CV measurements, this temperature dependence has been also observed in the impedance spectra measurements. Figure 3a shows the Nyquist plots—presenting the imaginary part, −*Z_imag_*, as a function of the real part, *Z_real_*, of the complex impedance in the temperature range investigated. As can be seen, the diameter of the semicircle loop reduces with the increase in the temperature. By taking into account the simplified ac R-C model described by Equation (2), the diameter can be associated with the interface resistance where faradaic reactions occur [33]. This means that the temperature variation produces some electron-transfer redox through reaction in the device.

**Figure 4:**
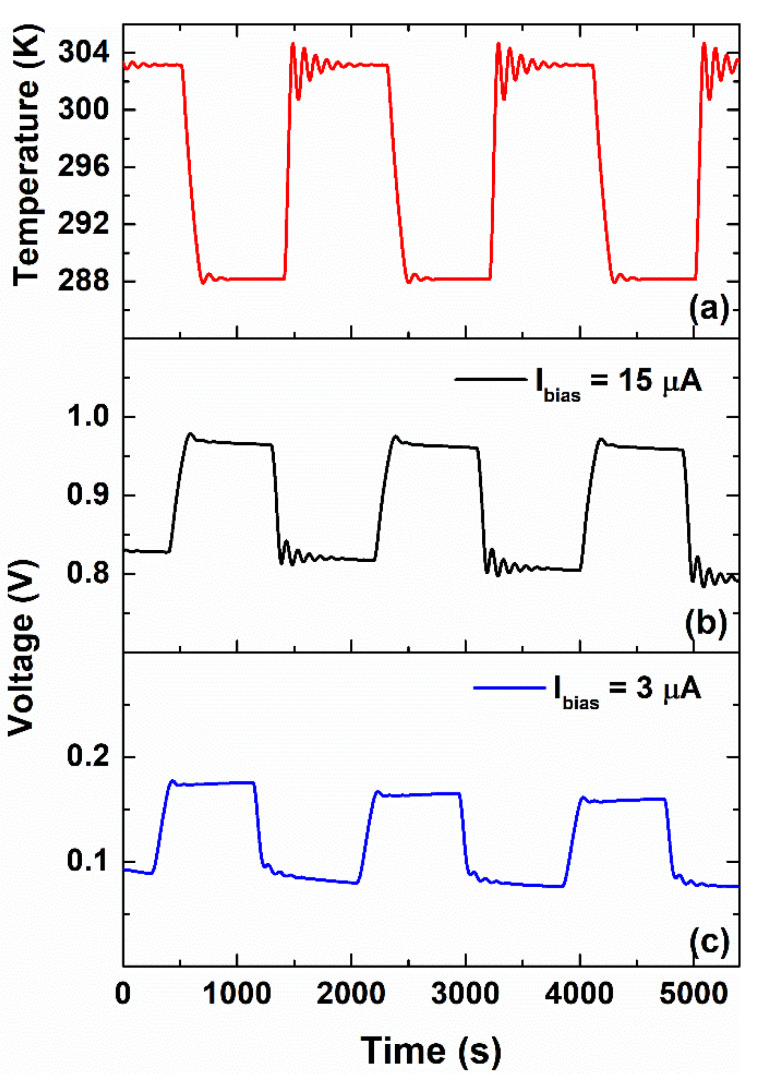
Time evolutions of the (**a**) square wave temperature profile imposed on the sample between 288 and 303 K and corresponding voltage signals, measured during the temperature stress, at bias current values of (**b**) 15 and (**c**) 3 μA, respectively. The visible “ringing” effect is due to the operation of the PID system and is not significative for the understanding of the system operation.

Figure 3b shows the temperature dependence of the ratio $C_{EIS}/C_{0}^{*}$, where $C_{EIS}$ is the capacitance extracted by using Equation (3) from the impedance data at a lower frequency (e.g., 0.1 Hz) and at open-circuit voltage. Here, $C_{0}^{*}$ corresponds to a reference value of $C_{EIS}$ at 288 K. At low frequency, the contribution of the interfacial charge accumulation to $C_{EIS}$ is dominant [34,35]. As evidenced, an increase in the temperature produces growth of the capacitance $C_{EIS}$. Silva et al. reported on a temperature sensor based on interdigital electrodes coated with gelatin solution with lower *TCC* values measured with EIS spectra (1.5 %/K at 800 MHz) [16]. The *T* variation also influences ion mobility within the gel nanocomposite. The temperature dependence of the ionic conductivity, computed by using Equation (4), is displayed in Figure 3c. As can be observed, the rise in the temperature leads to an increment of $\sigma_{ac}$ from 0.4 mS/cm^2^ at 288 K (15 °C) up to a value of 0.7 mS/cm^2^ at 303 K (30 °C). These values are in good agreement with what is found in the literature for gelatin-based polymer electrolyte [35,36]. Here, the gelatin is a protonic-conducting gel where the diffusion coefficient of the H^+^ ions, through hydrogen bonding between the amine and hydroxyl groups of the matrix, is higher than that observed for the divalent ions (i.e., Ca^2+^, Cu^2+^, and Fe^2+^) [32,37]. The presence of these latest ions in the blend originates from the denaturation process of the native collagen. The network of hydrogen bonds facilitates the long-range hopping motion of H^+^ which is activation energy-driven and, therefore, is highly dependent on temperature [4,35]. It should be noted that the gelatin and water-glycerol molecules are also mixed homogeneously, contributing to the formation of this ion-conducting path [38]. By increasing the temperature, the number of H-bonds within the biopolymer increases, leading to a variation in the charge carrier accumulation at the metal/gel–nanocomposite interface. As a consequence, the output electrical signal of the device changes. To investigate the influence of temperature on dielectric properties, the voltage response of the device has been measured during multiple thermal cycles under a bias current. Figure 4a shows the time evolution of a square wave temperature profile imposed on the sample between 288 and 303 K.

**Figure 5:**
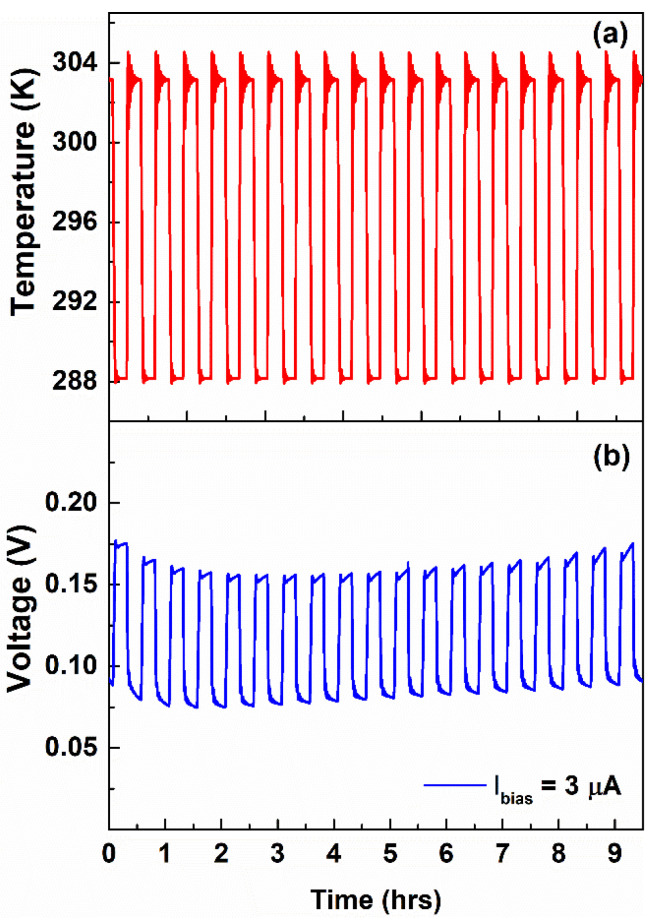

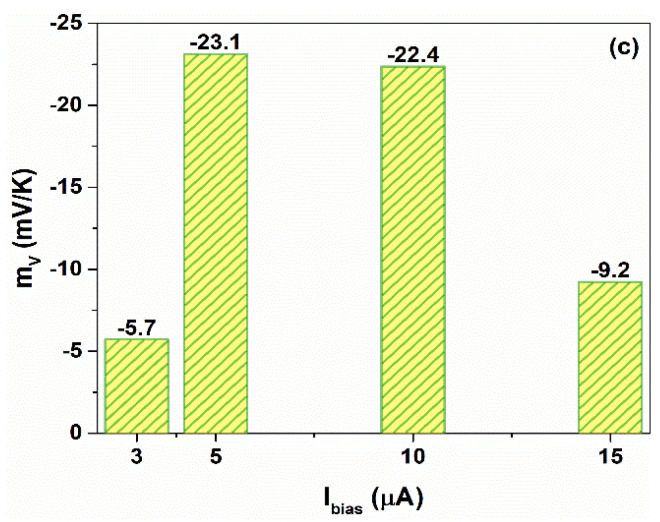
Time evolutions of the (**a**) square wave temperature profile imposed on the sample between 288 and 303 K during the cycling endurance test and (**b**) corresponding voltage signal measured at a bias current value of 3 μA, respectively. (**c**) Voltage temperature sensitivity of the device for different bias currents.

The corresponding voltage signals measured during the temperature stress, at a bias current value of 3 and 15 μA, are shown in Figure 4b,c, respectively. As can be seen, an increase in temperature leads to a decrease in the measured voltage signal. This behavior has been also observed for other bias current values (e.g., 5 and 10 μA), as shown in Figure S1 (Supplementary Materials). Here, the device detects the temperature variation and works as a temperature sensor having a negative dependence characterized by a voltage sensitivity $m_{V}=\Delta V/\Delta T$, where ΔV is the variation in the output electrical signal. Several authors report, in the literature, temperature sensors based on natural biopolymers, obtained from renewable resources (such as cellulose, silk, chitosan and tobacco cells) that are electrically biased [1,39] or self-powered [8] with a negative voltage sensitivity. In this latter case, the thermal-to-electric conversion mechanism is achieved through the thermogalvanic, or Soret, effect [34,40]. In our samples based on the gelatin, no reproducible results have been observed without applying current biasing. In Figure 5a,b, the time evolutions of the temperature response of the device measured for more than 9 h under operating conditions, corresponding to about 20 cycles, are shown.

As evidenced, the dielectric properties maintained during the endurance test and the voltage value measured at low and high-temperature regimes are about 159 mV and 80 mV, respectively. Therefore, the resulting $m_{V}$ has an average value of about −5.3 mV/K for the entire time of operating with $I_{bias}$ = 3 μA. Figure 5c displays the $m_{V}$ values as a function of the bias currents, evidencing that the voltage temperature sensitivity is current-dependent. As can be seen, during the current-controlled mode, the extracted $m_{V}$ is −5.7 mV/K at 3 μA, which increases up to a value of about −23 mV/K for $I_{bias}$ ranging between 5 and 10 μA and, subsequently, decreases down to −9 mV/K at 15 μA. These values have been extracted from the falling edges of the voltage profiles shown in Figure 4 and Figure S1 (Supplementary Materials). Since ΔT is the same during the temperature cycles, it can be assumed that the change in $m_{V}$ can be related solely to the bias current. It is worth noting that a change in $I_{bias}$ influences both the ion density accumulated at the metal/gel nanocomposite interface (e.g., electrode polarization) and also that involved in the faradaic processes within the electrochemical sensor.

### 3.2. Evidence of the Limiting Current Phenomena in the Gelatin Based-Electrolyte

**Figure 6:**
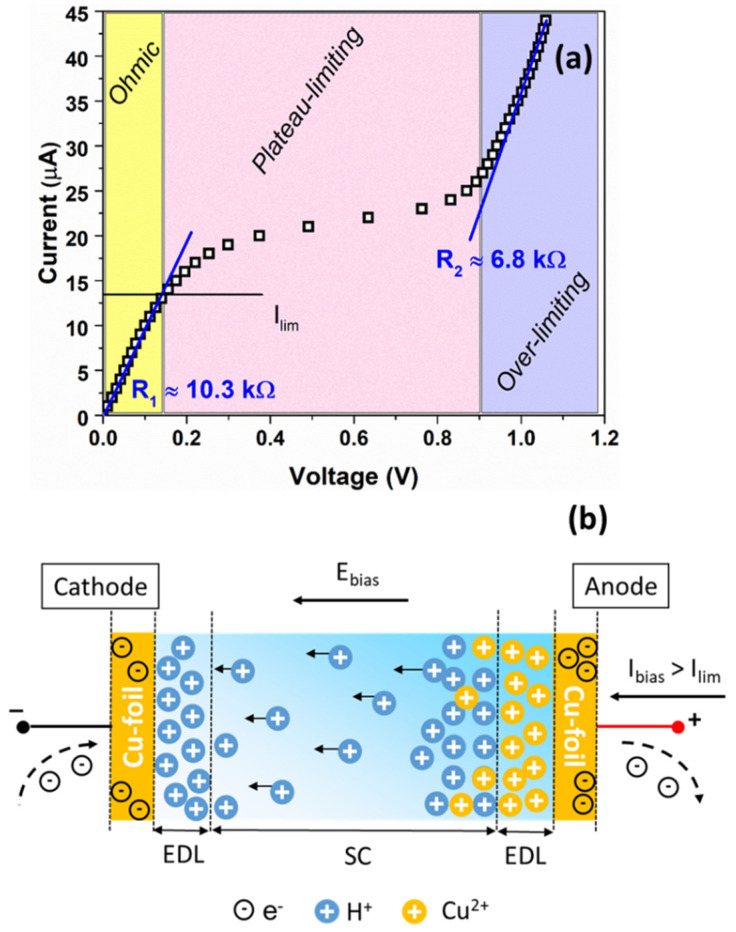
(**a**) Current–voltage characteristic of the temperature sensor under current biasing and (**b**) schematic illustration representing the charge accumulation and faradaic processes within the blend when $I_{bias}>I_{lim}$.

In order to evaluate the correlation between the ion distribution within the blend and the bias current that leads to a temperature-dependent voltage response of the sensor, a chronopotentiometry characterization has been performed. Here, the imposed current profile consists of a succession of step impulses of equal magnitude (1 μA) and that are spaced equally in time (60 s). Figure 6a displays the corresponding current–voltage characteristic showing three distinct regions denoted as ohmic, plateau-limiting and over-limiting. In the first region, the voltage signal increases linearly as a function of the current applied, showing a resistance $R_{1}\approx$ 10.3 kΩ. Conversely, in the plateau-limiting region, the current remains relatively constant while the voltage increases. This particular value refers to the limiting current which corresponds to the current value ($I_{lim}$) from which dissociation reaction within the device begins [41]. It should be noted that the biopolymer is water-processed material and contains a large amount of water-glycerol molecules trapped within the blend [12]. These molecules participate in the dissociation processes when $I_{bias}>I_{lim}$. The involved reactions lead to an increase in the ion concentration and justify the decreasing of the resistance $R_{2}\approx$ 6.8 kΩ measured in the over-limiting region. In order to identify the value of $I_{lim},$ a Cowan–Brown method, consisting of plotting the resistance $V/I_{bias}$ against the reciprocal current $1/I_{bias}$, has been applied [42]. Figure S2 (Supplementary Materials) shows the Cowan–Brown plot indicating that $I_{lim}$ has a value of 13 μA, consistent with what is found in Figure 6a. Under these operating conditions, the device works as an electrolytic cell.

For current values exceeding the limiting current, a non-spontaneous oxidation reaction of Cu/Cu^2+^ at the top copper electrode also occurs in the sensor. These ions accumulate in the electrode/gel nanocomposite interface and remain close to the electrical-double layer (EDL) under the anode [43]. It should be noted that these divalent ions have low mobility within the biopolymer and can form ionic bonds with the carboxylic acid groups on the gelatin polypeptides, influencing the organization and properties of the gelatin network (e.g., stability, mechanical strength and chemical crosslinking) [37]. In Figure 6b, a schematic illustration representing the charge accumulation and the faradaic charge transfer mechanisms within the temperature sensor under operating conditions is shown. As can be observed, the external electric field $E_{bias}$ imposes a potential gradient within the device thickness, leading to a drift of the cations to the negative electrode with consequent development of a further EDL at the cathode interface.

By assuming that the gelatin has a formula of NH_2_-X-COOH, where X is the main gelatin structure and COOH and NH_2_ are the acid and the basic groups, respectively, the alkali-conditioned treatment (resulting from the fabrication process) makes the pure gelatin positively charged and the obtained gel–nanocomposite acts as a weak acid. The biopolymer can be seen as a source of protons due to the protonation of both the carboxyl and amine groups NH_3_^+^-X-COOH [44]. Therefore, the H^+^ ions are the dominant cations within the space charge (SC) layer and reach the cathode thanks to the ion conduction mechanism assisted by the hydrogen bonds and by the electric field [45]. Here, the copper surface at the cathode terminal acts as an inert electrode on which the H^+^ ions accumulate. The reduction reactions occur within the gel–nanocomposite between proteins/peptides (e.g., functional groups) and the water–glycerol molecules. The measured potential is the sum of two interfacial potentials at the electrodes and any potential occurring across the gel–nanocomposite as current flows [43].

**Figure 7:**
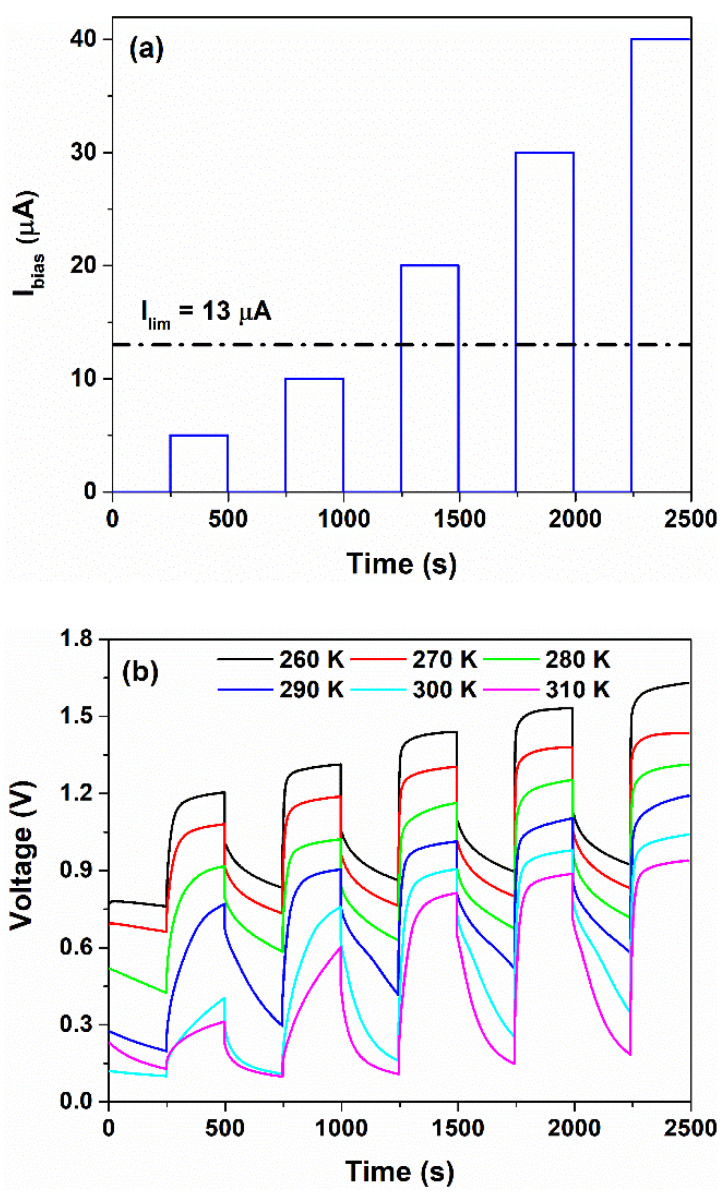
Time evolution of the (**a**) bias current profile and the (**b**) corresponding voltage measured across the device under operating conditions, respectively.

The time evolution of the bias current profile and the voltage measured across the device under operating conditions are shown in Figure 7a,b, respectively. In order to describe, in detail, the correlation between the temperature and the voltage response of the sensor, the ΔT value has been increased up to 50 K between 260 and 310 K (that is, −13 °C and +37 °C). As can be observed, when the device is biased, the voltage response is characterized by an initial jump due to the series resistance of the device and the subsequent increase in the voltage, reaching a saturation level $V_{sat}$. Conversely, when the bias current is switched off, the voltage signal decreases, which is caused by the discharge processes occurring within the device.

At lower temperatures (≤280 K), the measured voltage profiles follow a square wave for each bias current value. Conversely, at 290 K, the output voltage curve starts to differ from the square wave shape. This difference can be observed for $I_{bias}$ of 5 μA and 10 μA, as displayed in Figure 7b. Additionally, for higher temperatures (300 and 310 K), the triangle profiles become more evident for bias current values lower than the limiting current. In this latter case, the sensor works in an ohmic regime.

It seems that $I_{lim}$ shows a dependence on the temperature which influences the sensor response. As T increases, the limiting current increases and the plateau region, observed in Figure 6a, shifts toward higher voltage. When the temperature rises, the ionic conductivity of the blend increases due to an increment in the concentration of the protons within the blend released by the COOH and NH_3_^+^ groups, as evidenced by Figure 3c. Therefore, the $I_{lim}$ value, which is proportional to the concentration of the ions that participate in the dissociation reactions, increases as well. This trend is in good agreement with what is reported in the literature [38,46]. As a consequence, under operating conditions and for temperatures higher than 290 K, the sensor works properly for values of $I_{bias}$ at least 20 μA. Additionally, the speed response of the sensor is influenced by the bias current; for values lower than $I_{lim},$ the device shows a slow dynamic of tens of seconds. On the contrary, when the system works for $I_{bias}>I_{lim}$, the transients are faster. This means that the formation of the double layer at the electrode interface and the faradaic charge transfer mechanisms modifies the performances (e.g., speed, sensitivity, linearity) of the electrochemical sensor.

### 3.3. DC Electrical Model for the Temperature Sensor under Current-Control

**Figure 8:**
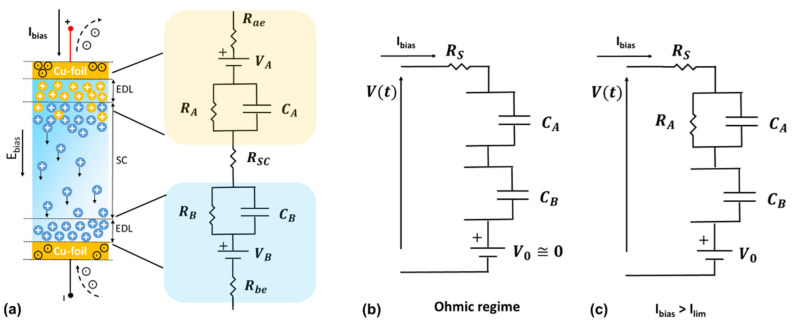
(**a**) Physical representation (from Figure 6b) and the corresponding DC equivalent circuit illustrating the ion accumulation, the electrode potential and the faradaic reactions involved in the device. (**b**,**c**) Simplified versions of DC circuit for the temperature sensor under current-controlled mode in ohmic regime and with $I_{bias}>I_{lim}$, respectively.

In order to quantify the mechanisms involved in temperature sensing, the equivalent electrical DC model has been proposed. In Figure 8, the equivalent circuit describing the charge carrier accumulation, the faradaic charge transfer and diffusion processes within the device under the current-controlled (also called galvanostatic) method are shown.

When a current control signal is applied and the threshold current ($I_{lim}$), at which the reactions typically start, is not yet reached, the sensor works in the ohmic regime. As a consequence, the electrical DC model is simply composed as a series connection between a resistance (accounting the ohmic contributions of the blend) and the capacitive contribution of the electrode interfaces. In this case, the interfaces metal/gel nanocomposite can be considered as an ideally polarizable electrode and, therefore, only a capacitive (non-faradaic) current is flowing in $C_{A}$ and $C_{B}$, respectively [43]. The simplified version of the circuit is shown in Figure 8b. It is worth noting that the capacitance takes into account the accumulation of ions at an electrical double layer, whereas the faradaic current accounts for electron-transfer via redox reactions at the electrodes. This latter contribution is modeled as a further parallel resistance to the double layer capacitance. For $I_{bias}>I_{lim}$, the faradaic currents start passing through both $R_{A}$ and $R_{B}$ in the branches of the circuit parallel to $C_{A}$ and $C_{B}$, respectively, as shown in Figure 8a. The electrode potentials that have arisen from the oxidation reaction at the anode and reduction reaction within the blend close to the cathode are represented by $V_{A}$ and $V_{B}$, respectively [43]. Under the galvanostatic mode, the electrochemical temperature sensor can be modeled as Figure 8c. Here, $R_{S}$ takes into account both the ohmic contribution of the space charge layer of the blend and of the electrodes. Since the cathode works as inert electrode, no faradaic reactions occur at the interface. Therefore, it can be modeled as a capacitor $C_{B}$ that accounts for the charges within the diffuse layer and at the metal interface.

**Figure 9:**
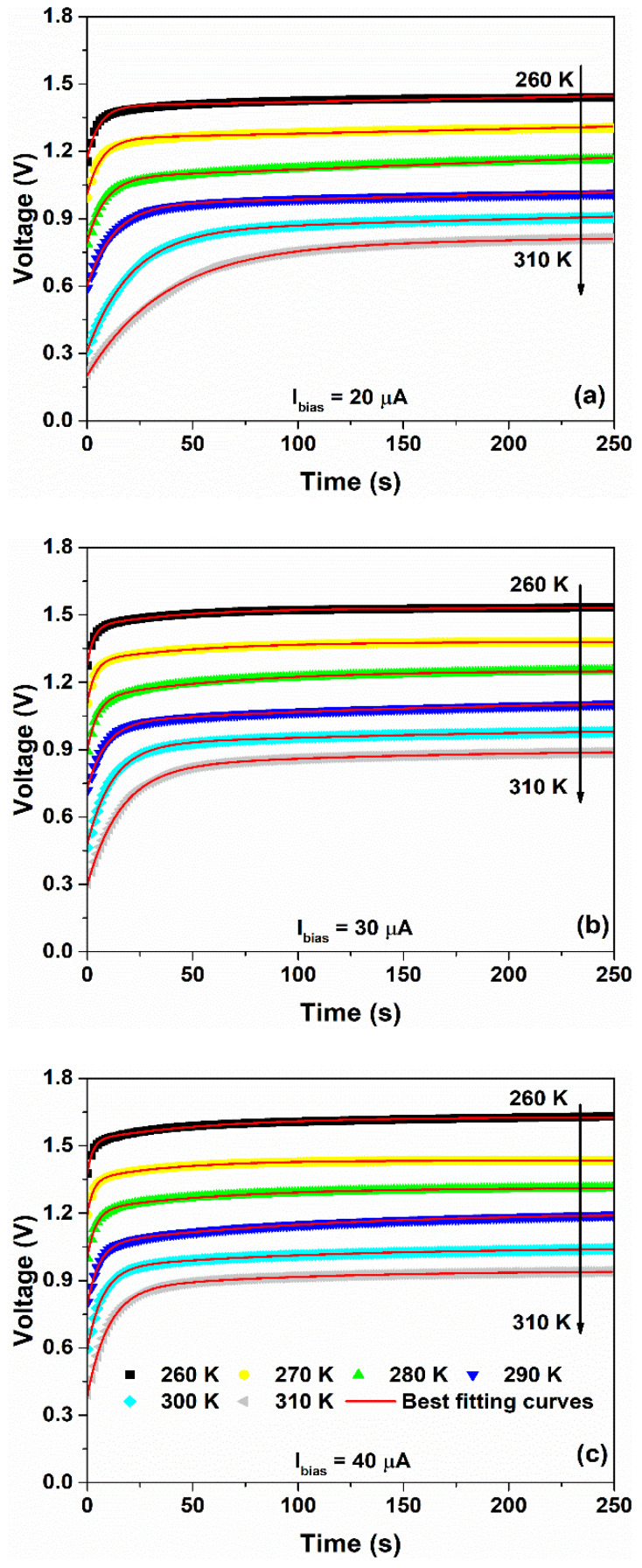
Time evolution of the voltage measured across the device under operating conditions at (**a**) 20 μA, (**b**) 30 μA and (**c**) 40 μA as a function of the temperature between 260 and 310 K, respectively. The best fitting curves, shown as red solid lines, are obtained from Equation (5).

In this framework, the output voltage V(t) under bias current can be written as

(5) $$V\left(t\right)=V_{0}+R_{S}I_{bias}+R_{A}I_{bias}\left(1-e^{-\frac{t}{\tau_{A}}}\right)+\frac{I_{bias}}{C_{B}}t\to ,$$

where t is the time in seconds, $V_{0}=V_{B}-V_{A}$ is the open circuit potential and $\tau_{A}=R_{A}C_{A}$ is the time constant associated with the anode kinetics. This latter term influences the speed response of the sensor. It is worth noting that when $I_{bias}=0,$ the device works as a galvanic cell and the observed open-circuit voltage $V_{0}$ is related to variation in temperature of the redox activity [43]. Figure 9 shows the time evolutions of the voltage measured across the sensor for $I_{bias}>I_{lim}$ and for temperatures between 260 and 310 K.

**Figure 10:**
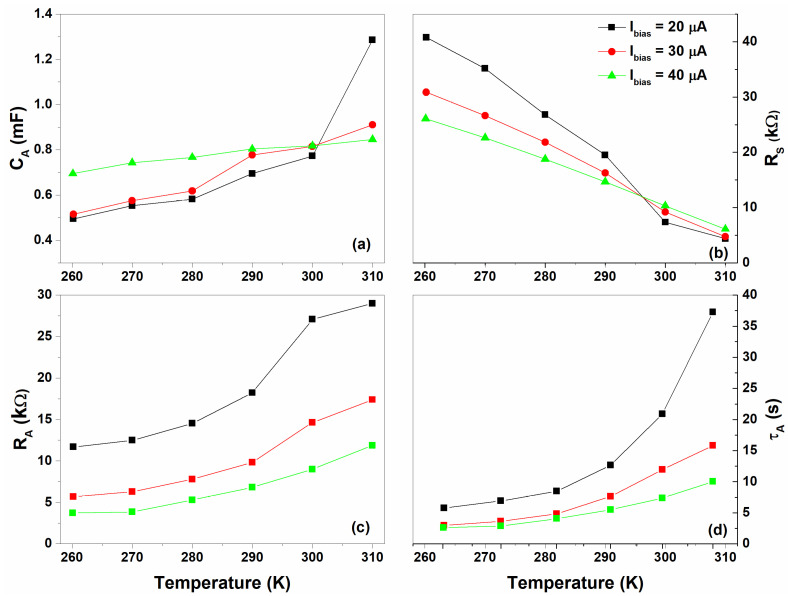
Temperature dependence of the (**a**) capacitance $C_{A}$, (**b**) series resistance $R_{S}$, (**c**) faradaic resistance $R_{B}$ and (**d**) time constant $\tau_{A}$ computed from the fitting procedure at bias current range between 20 and 40 μA, respectively.

The theoretical model of Equation (5) has been used to fit the experimental data and is depicted in Figure 9 as red solid curves. The resulting best fitting parameters are shown in Figure 10. Here, an increase in the temperature leads to an increase in the ion concentration within the gel–nanocomposite and, therefore, an increment of the ion conductivity within the sensor.

As can be seen in Figure 10a, the capacitance related to the anode ($C_{A}$) shows a positive temperature coefficient in good agreement with what is reported in Figure 2c. Additionally, the reduction in $R_{S}$ as a function of the temperature, observed for all the bias current values in Figure 10b, can be related to the rise of ion conductivity. Conversely, the resistance $R_{A}$, which takes into account the faradaic processes within the sensor, slightly increases with the temperature (see Figure 10c). For $I_{bias}>I_{lim}$, the sensor acts as an electrolytic cell, where the exchange current, involved in the faraday reactions, decreases with the temperature. It should be noted that the $R_{A}$ values decrease as the bias current increases, proving that these reactions are governed by Faraday’s law (i.e., the amount of chemical reaction is proportional to the bias current) [47]. As a consequence, the time constant $\tau_{A}=R_{A}C_{A}$ shows an exponential growth relation with the temperature reaching values ranging between a few seconds to tens of seconds (see Figure 10c). Here, the temperature dependence comes from the electrochemical processes (e.g., diffusion-limited processes) occurring within the sensor in the limiting-current region. This range of response times is in good agreement with what is reported in the literature for electrochemical devices [8,9,48].

By taking into account the Equation (5), after a sufficiently long time $\Delta \tau \approx 4\tau_{A}$, the voltage response of the sensor reaches a saturation level of Vsat≈V0+Ibias(RS+RA+ΔτCB). Being ΔτCB≪(RS+RA), varying from 1.2 kΩ at 260 K to 0.8 kΩ t 310 K for $I_{bias}$ of 40 μA, the voltage sensitivity ΔV/ΔT of the sensor can be expressed as

(6) $$m_{V}=\frac{\Delta V_{0}}{\Delta T}+I_{bias}\left(\frac{\Delta R_{S}}{\Delta T}+\frac{\Delta R_{A}}{\Delta T}\right),$$

where $m_{o}=\frac{\Delta V_{0}}{\Delta T}$ is the temperature variation in the open-circuit voltage and $m_{R}=\left(\frac{\Delta R_{S}}{\Delta T}+\frac{\Delta R_{A}}{\Delta T}\right)$ is the voltage temperature variation in the resistances related to the faradaic processes and to the space charge layer under current biasing. It should be noted that for the whole temperature range investigated, the quantity $\left(R_{S}+R_{A}\right)$ decreases with the temperature, as displayed in Figure S3 (Supplementary Materials).

**Figure 11:**
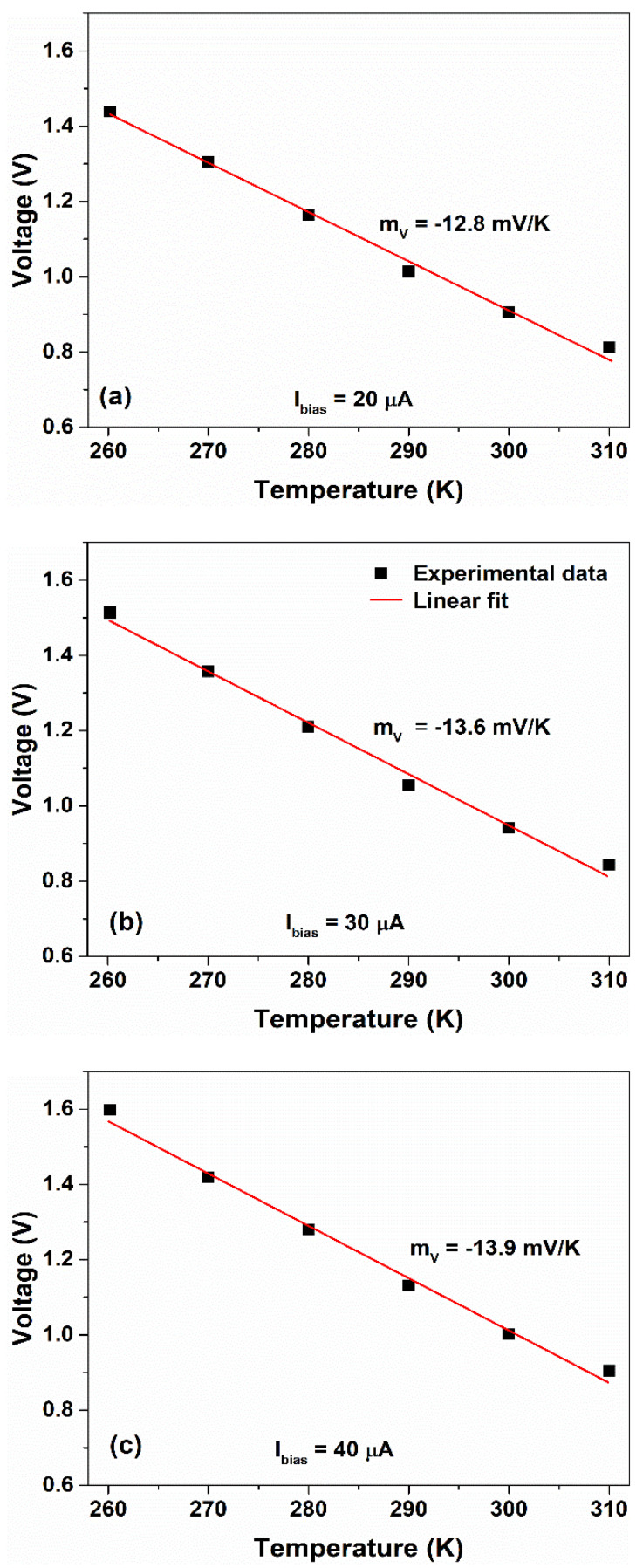
Variation in the voltage measured across the sensor as a function of the temperature for bias current values of (**a**) 20 μA, (**b**) 30 μA and (**c**) 40 μA, respectively. The red solid lines represent the linear fit.

In Figure 11, the voltage sensitivity values, extracted from the experimental data at different values of $I_{bias}>I_{lim}$, have been shown. As can be seen, the sensor shows a linear voltage sensitivity $m_{V}$, having an average value of (−13.4±0.5) mV/K corresponding to an average percentage variation $m_{V}/V^{*}$ of − 0.9%/K, where $V^{*}$ is the voltage at 260 K.

**Figure 12:**
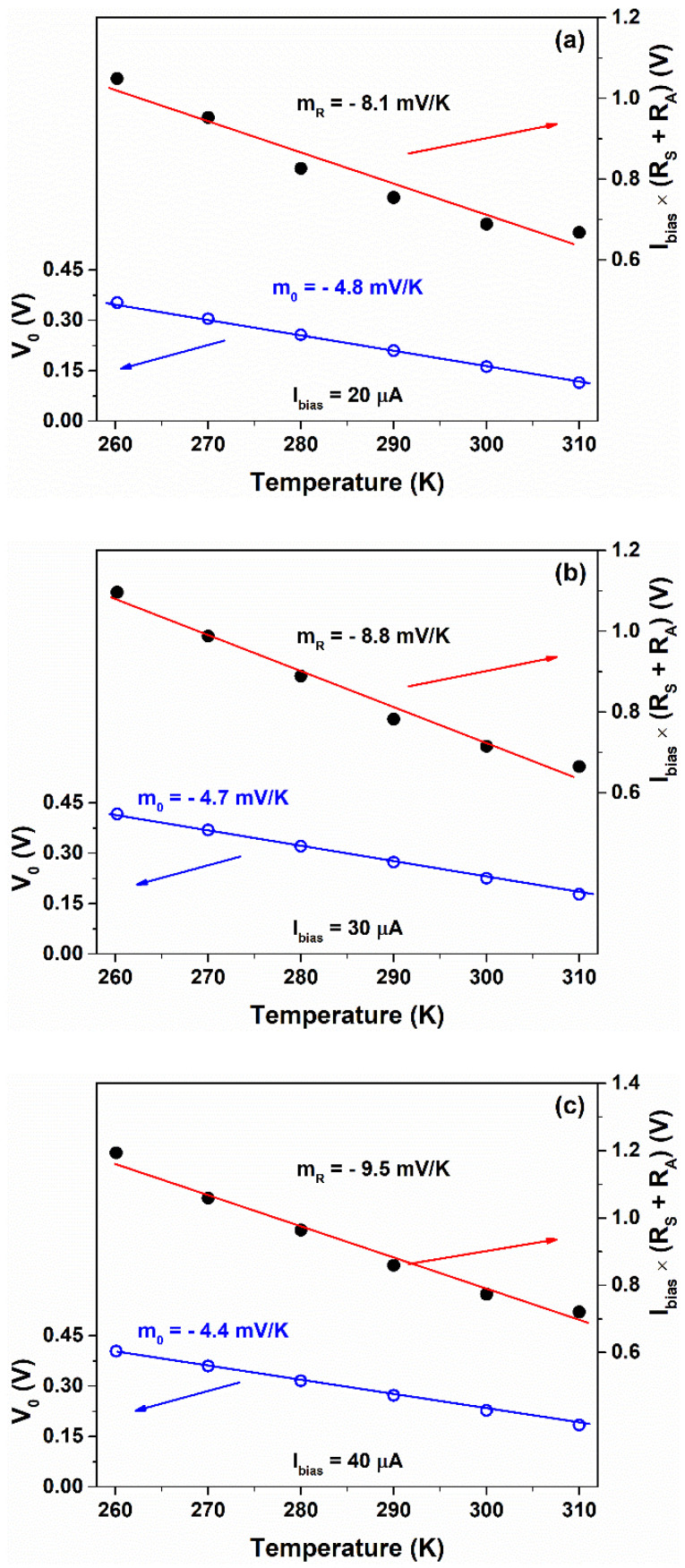
Partition method applied to the voltage measured across the sensor as a function of the temperature for bias current values of (**a**) 20 μA, (**b**) 30 μA and (**c**) 40 μA, respectively. The red and blue solid lines represent the linear fit for the self-powered and current biased components, respectively.

Similar to Equation (6), the voltage sensitivity is: $m_{V}=m_{0}+I_{bias}\;m_{R}$, where $m_{0}$ and $m_{R}$ contributions can be computed from the best fits. Figure 12 shows the contributions to the sensitivity $m_{V}$ of the self-powered ($m_{0}$) and current biased ($m_{R}$) components extracted from the voltage response for bias current values ranging between 20 and 40 μA. As evidenced, the resulting values estimated by the linear fits are consistent with the voltage sensitivities reported in Figure 11.

Here, the surface potential $V_{o}$ of metal electrodes varies with temperature through the variations in the ion concentration in the electric double-layer at the metal/gel–nanocomposite interface. This effect has been already reported in the literature as the main thermal sensing mechanism for self-powered sensors and thermally chargeable supercapacitors [8,49]. Although, in the ohmic regime, the sensor is able to detect the temperature variation (as shown in Figure S4 of the Supplementary Materials), our samples are not able to reproduce temperature steps without current biasing. Here, the resulting $m_{o}$ values are consistent with what is reported in the literature for a symmetric electrolyte-assisted temperature sensor [8].

For $I_{bias}<I_{lim}$, the device shows a non-constant value of $m_{V}$ as a function of the temperature (see Figure S5 of the Supplementary Materials). Here, the electrochemical device works in the ohmic regime, showing a non-linearity of sensitivity and slower speed response. This finding is evident in the corresponding voltage responses (Figure S4 of the Supplementary Materials), where triangle shapes in the output voltage are present. In Tables S1–S7 (Supplementary Materials), the performance data of the sensor are listed in terms of speed response, power consumption and voltage sensitivity as a function of the bias currents. Although the sensing mechanism is assisted by the ions within the gelatin nanocomposite, the electrochemical sensor shows a response time (to reach the saturation value) ranging between 23.1 s at 260 K and 149 s at 310 K, with a power consumption range between 0.14 and 0.61 mW at 20 μA, respectively. Since the speed response is influenced by the bias current value, in order to improve the response time, it is necessary to increase the bias’ current value. At 40 μA, the device shows the fastest response time, ranging between 10.4 s at 260 K and 40 s at 310 K, with a power consumption range between 0.17 and 0.38 mW. The response time of the temperature sensor is lower than that reported in the literature (150–220 s) for the self-generated device based on a gel-like electrolyte [8]. It should be noted that during the measurement, the device has a few tenths of milli-watts of power consumption. This low value is obtained because the sensor, under operating conditions, takes advantage of a self-generated ($V_{0}$) portion of energy.

### 3.4. Driving Circuit for Reducing the Voltage Drift and Offset of the Temperature Sensor

**Figure 13:**
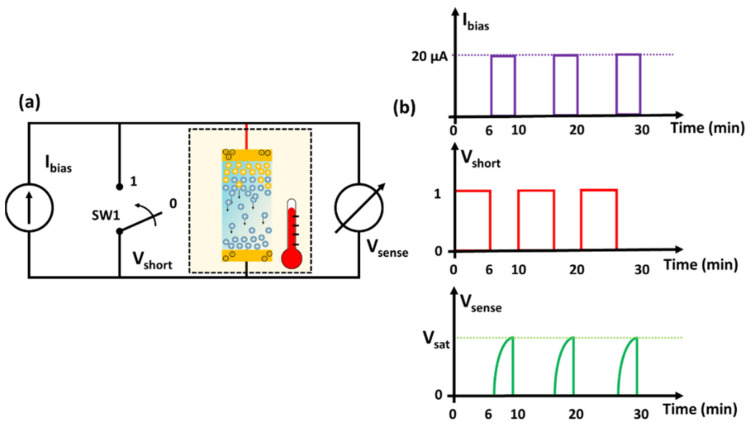
(**a**) Schematic circuit used for the measurements. The part inside the dashed box is temperature controlled. (**b**) Typical time sequence of the bias current, switch state and output signal.

**Figure 14:**
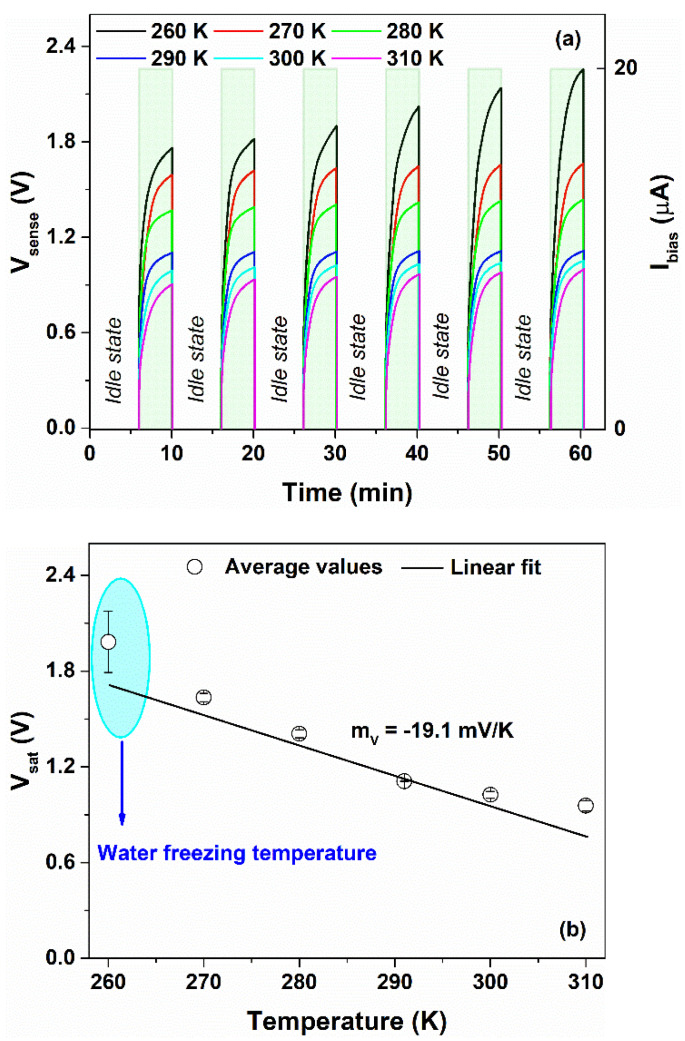
(**a**) Time evolution of the voltage across the device (y-left axis) and corresponding bias current profile (y-right axis), measured by using the logic circuit. (**b**) Variation in the output voltage as a function of the temperature for bias current of 20 μA. The solid line represents the linear fit.

The output voltage of the device suffers a drift of the $V_{sat}$ value when it is in the on state ($I_{bias}\neq 0$). Additionally, when the sensor is in the off state ($I_{bias}=0$), the output voltage is driven by the self-discharge processes occurring within the device that are not fully controlled. In particular, they depend on the history of the device (e.g., electrode potentials and charges stored in the system before the discharge) and on the measurement conditions (e.g., operating time and bias currents). As a consequence, for each measurement cycle, a memory effect on the voltage response can be observed that shows a difference in the offset values, as shown in Figure 7b. This drift causes less accuracy in the temperature measurements. In order to reduce this error contribution, a simple bias circuit has been designed. Figure 13a,b shows the implemented logic circuit and the corresponding signals, respectively. When the sensor is switched off, the voltage across the device is shorted electrically by using the switch $SW_{1}$ controlled by the signal ($V_{short}$). Under these operating conditions, the sensor is forced to quickly deplete the charge accumulated within the device and reaches a reproducible idle state. Subsequently, when the switch $SW_{1}$ is open, the bias current flows in the sensor and the device goes into sensing mode: the voltage $V_{sense}$ increases, coming to a saturation value. The proposed circuit is designed to perform one measurement every 10 min with an idle time of 6 min and a sense period $\tau_{sense}$ of 4 min. The corresponding voltage responses of the sensor, resulting from six continuous measurement cycles in a temperature range between 260 K and 310 K at 20 μA, are shown in Figure 14a. As can be noted, the sensor recovers its open-circuit voltage after being electrically shorted in the idle time. The use of the circuit prevents the unregulated discharge phenomena and leads to a better reproducibility of the response for the whole the temperature range in sensing mode. The observed drift effect from the curves in Figure 7b disappears and the saturation level of the output voltage is more stable during the cycling measurements. Conversely, at 260 K (−13 °C), the voltage response, after an operating time of 30 min, increases as a function of the time, and a slight drift has been noted. This behavior can be related to the water freezing within the gel–nanocomposite caused by the subzero temperatures.

Although the glycerol forms hydrogen bonds with the water molecules which can effectively prevent the freezing of the hydrogel, after an extended time at subzero temperatures, the blend becomes more rigid and less conductive [19,50]. As a consequence, the voltage signal increases at each cycle during the sensing. It is worth noting that the sensor can detect the ice/frost with an increase in the output voltage by changing 25% compared to its initial value. During the sensing mode, the energy consumption $E\approx V_{sense}I_{sense}\tau_{sense}$, when $V_{short}\approx 0$ and $I_{sense}$ = 20 μA, needed to power the sensor by using the driving circuit varies between 6.7 and 13.6 μWh at 260 and 310 K, respectively. The values computed for each temperature are listed in Table S8 (Supplementary Materials). The low values of energy consumption permit the use of the sensor in combination with energy storage systems (e.g., batteries and supercapacitors) that can keep the sensor working when the primary energy sources are not usable [39]. This could enable optimization of the energy efficiency of the sensor node. It is worth noting that to guarantee the sustainability of the systems, environmentally-friendly energy devices can be used [22,33].

In Figure 14b, the voltage measured across the sensor as a function of the temperature is shown. The use of the logic circuit that controls the discharge phenomena within the device leads to an increment in the sensitivity, reaching a value of −19.1 mV/K. This value is higher than that reported in the literature (about −11 mV/K) for a self-powered temperature sensor based on a gel-like electrolyte with a least eco-friendly ionic liquid [8]. At 260 K, the slight increase in the voltage response, caused by the water freezing temperature, leads to an increment in the degree of spread in the experimental dataset, as indicated by the error bar in Figure 14b. Additionally, the sensor shows an output signal with zero-drift of the voltage, without offset component and higher cycle stability compared to the signal measured previously.

## 4. Conclusions

An environmentally-friendly temperature sensor has been fabricated by using a low-cost water-processable nanocomposite material based on gelatin and graphene. The temperature dependence of the electrochemical properties has been investigated by using cyclic voltammetry, chronopotentiometry and impedance spectroscopy measurements. The symmetric device, composed of a sandwich structure between metal foils and a printable graphene–gelatin blend, exhibits a temperature dependence of the open-circuit voltage in a range between 260 and 310 K.

To ensure an environmentally safe and easily disposable device, any ionic components that may adversely affect the environment have been avoided. Here, the gelatin acts as a binder for holding together the hydrophobic graphene filler with the water–glycerol molecules and as a solid electrolyte, being a protonic conductor. Moreover, the addition of glycerol, which forms hydrogen bonds with the water molecules, serves as an anti-freezing agent. The thermally-induced phenomena occur at the electrode/gel interface with a bias current of a few tens of μA. The occurrence of dissociation reactions within the sensor causes limiting-current phenomena in the gelatin electrolyte. A detailed model, describing the charge carrier accumulation, the faradaic charge transfer and diffusion processes within the device under the current-controlled condition, has been proposed. The voltage sensitivity can be expressed as the sum of the contributions related to the self-power and the current biasing component. Although the sensing mechanism is assisted by the ions within the hydrogel based on the gelatin, the electrochemical sensor shows a fast response time (to reach the saturation value) ranging between 10.4 s at 260 K and 40 s at 310 K, respectively.

In order to increase the cycle stability of the temperature sensor and reduce its voltage drift and offset of the output electrical signal, a driving circuit has been designed. The eco-friendly sensor shows a temperature sensitivity of about −19 mV/K, long-term stability and low-power consumption, in the range of microwatts, suitable for zero-waste environmental monitoring for indoor applications. At subzero temperatures, the device is able to detect the ice/frost formation.

## Data Availability

The data presented in this study are available on request from the corresponding author.

## Supplementary Materials

The following supporting information can be downloaded at: https://www.mdpi.com/article/10.3390/nano12132227/s1, Figure S1: Time evolutions of the temperature profile imposed to the sample and of the measured voltage signal; Figure S2: Determination of the limiting current value by using the method of Cowan and Brow; Figure S3: Variation of the sum of ohmic contributions $R_{S}+R_{A}$ extracted from the fitting procedure as a function of the temperature for bias current values (a) lower and higher than the limiting current of 13 μA, respectively; Figure S4: Time evolution of the voltage measured across the device in different operating conditions; Figure S5: Temperature dependence of the voltage measured across the sensor for different bias current values; Table S1: Device performances as a function of the bias current at 260 K; Table S2: Device performances as a function of the bias current at 270 K; Table S3: Device performances as a function of the bias current at 280 K; Table S4: Device performances as a function of the bias current at 290 K; Table S5: Device performances as a function of the bias current at 300 K; Table S6: Device performances as a function of the bias current at 310 K; Table S7: Device sensitivity as a function of the bias current; Table S8: Energy consumption as a function of the temperature.
