# Supplementary material for: Low-Power and Eco-Friendly Temperature Sensor Based on Gelatin Nanocomposite
Source: Nanomaterials (Basel). 2022 Jun 29;12(13):2227. doi: 10.3390/nano12132227 (PMC9268468; doi:10.3390/nano12132227)
Supplement: Supplementary file 1 [file nanomaterials-12-02227-s001.zip › nanomaterials-1771735-supplementary.pdf]

nanomaterials

Supplementary Materials

Low-Power and Eco-Friendly Temperature Sensor Based on Gelatin Nanocomposite

Giovanni Landi [1,*], Veronica Granata [2,3], Roberto Germano [4], Sergio Pagano [2,3,5] and Carlo Barone [2,3,5,*]

1 ENEA, Casaccia Research Center, Via Anguillarese 301, 00123 Rome, Italy
2 Dipartimento di Fisica "E.R. Caianiello", Università degli Studi di Salerno, 84084 Fisciano, SA, Italy; vgranata@unisa.it (V.G.); spagano@unisa.it (S.P.)
3 INFN Gruppo Collegato di Salerno, c/o Università degli Studi di Salerno, 84084 Fisciano, SA, Italy
4 PROMETE Srl, CNR Spin off, P.le V. Tecchio, 45, 80125 Naples, Italy; germano@promete.it
5 CNR-SPIN, c/o Università degli Studi di Salerno, 84084 Fisciano, SA, Italy
* Correspondence: giovanni.landi@enea.it (G.L.); cbarone@unisa.it (C.B.)

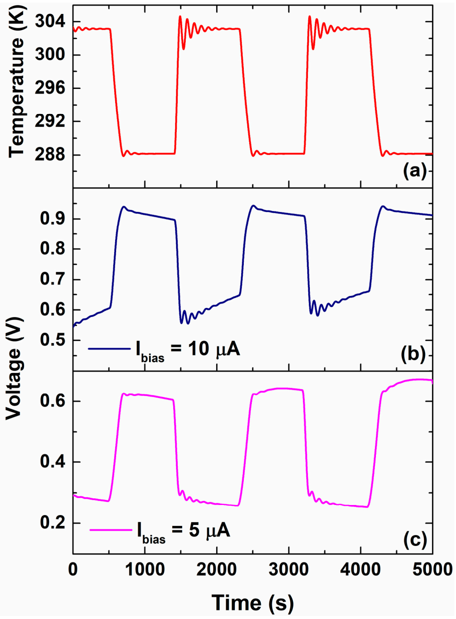

Figure S1. Time evolutions of the (a) square wave temperature profile imposed to the sample between 288 and 303 K and corresponding voltage signals, measured during the temperature stress, at a bias current values of (b) 10 and (c) 5 μA, respectively.

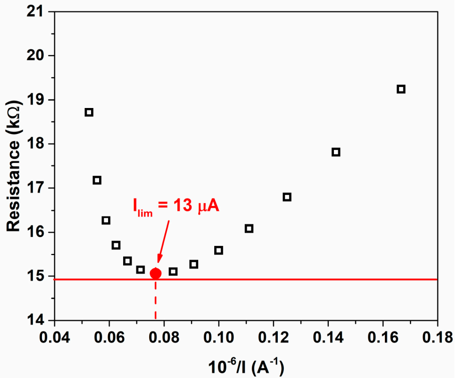

Figure S2. Determination of the limiting current value by using the method of Cowan and Brow.

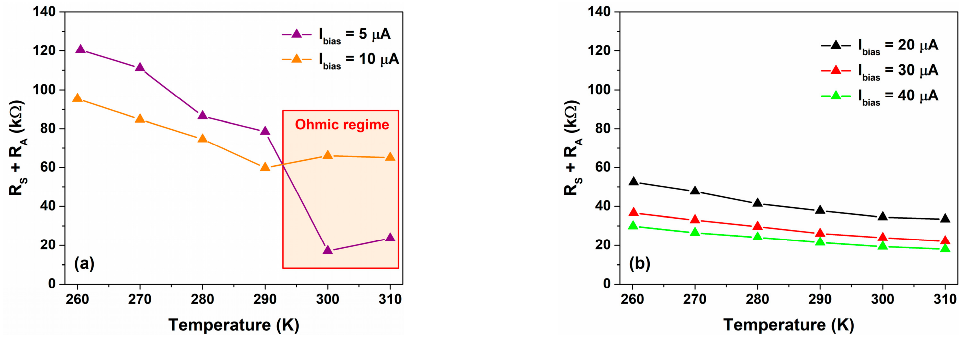

Figure S3. Variation of the sum of ohmic contributions $R_S + R_A$ extracted from the fitting procedure as a function of the temperature for bias current values (a) lower and higher than the limiting current of 13 µA, respectively.

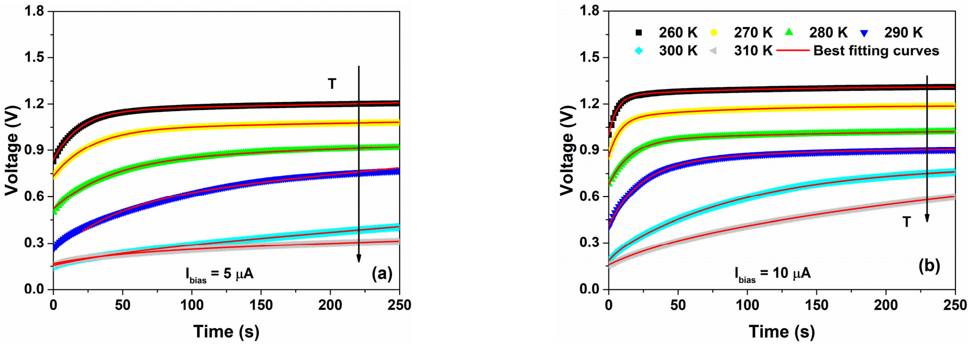

Figure S4. Time evolution of the voltage measured across the device under operating conditions at (a) 5 µA and (b) 10 µA as a function of the temperature between 260 and 310 K, respectively. The best fitting curves, using equation (5), are shown as red solid lines.

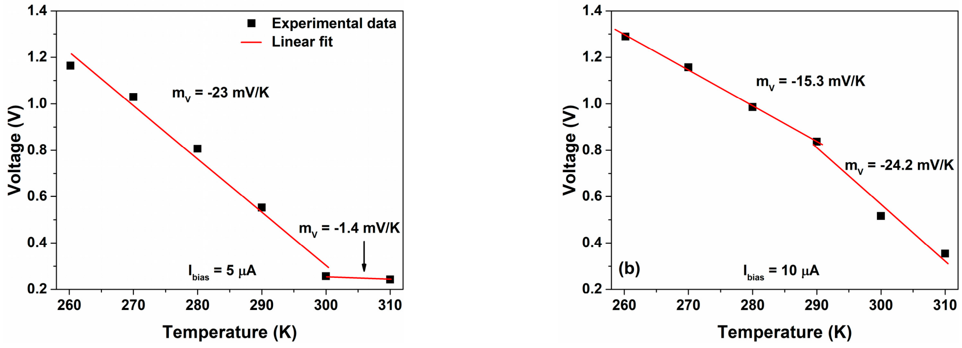

Figure S5. Variation of the voltage measured across the sensor as a function of the temperature for bias current values of (a) 5 µA and (b) 10 µA, respectively. The red solid lines represent the linear fit.

Table S1. Device performances as a function of the bias current at 260 K.

**Bias current (μA)**	**Speed Response Δτ (s)**	**Power consumption (mW)**
5(*)	74.5	0.11
10(*)	24.5	0.08
20	23.1	0.17
30	11.8	0.14
40	10.4	0.17

* Temperature sensor in ohmic regime.

Table S2. Device performances as a function of the bias current at 270 K.

**Bias current (μA)**	**Speed Response Δτ (s)**	**Power consumption (mW)**
5(*)	111.6	0.15
10(*)	31.6	0.09
20	27.6	0.18
30	14.5	0.15
40	11.5	0.16

* Temperature sensor in ohmic regime.

Table S3. Device performances as a function of the bias current at 280 K.

**Bias current (μA)**	**Speed Response Δτ (s)**	**Power consumption (mW)**
5(*)	167.0	0.19
10(*)	63.0	0.16
20	33.9	0.20
30	19.3	0.18
40	16.3	0.21

* Temperature sensor in ohmic regime.

Table S4. Device performances as a function of the bias current at 290 K.

Bias current (μA)	Speed Response Δτ (s)	Power consumption (mW)
5(*)	184.0	--
10(*)	84.2	0.19
20	50.6	0.26
30	30.6	0.25
40	21.9	0.26

* Temperature sensor in ohmic regime.

Table S5. Device performances as a function of the bias current at 300 K.

Bias current (μA)	Speed Response Δτ (s)	Power consumption (mW)
5(*)	214.9	--
10(*)	370.6	0.70
20	83.7	0.38
30	47.7	0.35
40	29.5	0.31

* Temperature sensor in ohmic regime.

Table S6. Device performances as a function of the bias current at 310 K.

Bias current (μA)	Speed Response Δτ (s)	Power consumption (mW)
5(*)	202.6	--
10(*)	755.4	1.13
20	149.1	0.61
30	63.3	0.42
40	40.2	0.38

* Temperature sensor in ohmic regime.

Table S7. Device sensitivity as a function of the bias current values.

**Bias current (µA)**	**Sensitivity (mV/K)**
5(*)	[-23, -1.4]
10(*)	[-24.2, -15.3]
20	-12.8
30	-13.6
40	-13.9

* Temperature sensor in ohmic regime.

Table S8. Energy consumption as a function of the temperature.

**Temperature (K)**	**Energy (*) (µWh)**
260	13.6
270	11.2
280	10
290	8.1
300	7.4
310	6.7

(*) Bias current of 20 µA.
